# Therapeutic targeting of HMGB1 during experimental sepsis modulates the inflammatory cytokine profile to one associated with improved clinical outcomes

**DOI:** 10.1038/s41598-017-06205-z

**Published:** 2017-07-19

**Authors:** Natalie E. Stevens, Marianne J. Chapman, Cara K. Fraser, Tim R. Kuchel, John D. Hayball, Kerrilyn R. Diener

**Affiliations:** 1 Experimental Therapeutics Laboratory, Hanson Institute, and Sansom Institute for Health Research, School of Pharmacy and Medical Science, University of South Australia, Adelaide, SA Australia; 2 Intensive Care Unit, Royal Adelaide Hospital, Adelaide, SA Australia; 3 Discipline of Acute Care Medicine, The University of Adelaide, Adelaide, SA Australia; 4 Preclinical, Imaging and Research Laboratories, South Australian Health and Medical Research Institute, Gilles Plains, Adelaide, SA Australia; 5 Robinson Research Institute, Adelaide Medical School, The University of Adelaide, Adelaide, SA Australia

## Abstract

Sepsis remains a significant health burden and a major clinical need exists for therapeutics to dampen the excessive and uncontrolled immune activation. Nuclear protein high mobility group box protein 1 (HMGB1) is released following cell death and is a late mediator in sepsis pathogenesis. While approaches targeting HMGB1 have demonstrated reduced mortality in pre-clinical models of sepsis, the impact of HMGB1 blockade on the complex septic inflammatory milieu and the development of subsequent immunosuppression remain enigmatic. Analysis of plasma samples obtained from septic shock patients established an association between increased HMGB1 and non-survival, higher APACHE II scores, and increased pro-inflammatory cytokine responses. Pre-clinically, administration of neutralising ovine anti-HMGB1 polyclonal antibodies improved survival in murine endotoxaemia and caecal ligation and puncture-induced sepsis models, and altered early cytokine profiles to one which corresponded to patterns observed in the surviving patient cohort. Additionally, anti-HMGB1 treated murine sepsis survivors were significantly more resistant to secondary bacterial infection and exhibited altered innate immune cell phenotypes and cytokine responses. These findings demonstrate that anti-HMGB1 antibodies alter inflammation in murine sepsis models and reduce sepsis mortality without potentiating immunosuppression.

## Introduction

Sepsis is characterised by an exacerbated inflammatory response following infection that damages host organs^1^. Once considered a syndrome of excessive inflammation, sepsis is now recognized as a syndrome of dysregulated immune function^2, 3^. While improved treatment paradigms have increased short-term survival from sepsis, an increased number of patients now experience prolonged immunosuppression which often culminates in long-term morbidity and mortality^4^. Consequently, novel treatment strategies should not only aim to increase short-term survival by controlling the acute inflammatory phase of disease, but also prevent long-term disability by restoring immune balance and function.

While dysregulated expression of the cytokines interleukin 6 (IL-6)^5^, tumour necrosis factor alpha (TNFα), IL-1 and IL-8^6^ has been associated with sepsis mortality, no therapeutic intervention targeting an individual cytokine has conferred significant benefit above standard monitoring and supportive care. A meta-analysis of TNFα-targeted therapies revealed only a 2% improvement to mortality compared to placebo^7^. Similarly, IL-1 receptor agonist (IL-1RA) administration exhibited limited clinical success^8^. The failure of such monotherapies has been linked to the timing of administration as expression of these cytokines mainly occurs during the ‘early-phase’ of disease, and therefore treatment may have been initiated too late. Administration of anti-inflammatory IL-10 has shown promise in reducing morbidity in preclinical sepsis models^9^, however additional IL-10 may promote secondary infection, as IL-10 has also been associated with post-septic immunosuppression^10^. Thus approaches targeting ‘late-phase’ mediators or master regulators of inflammation may hold more therapeutic promise.

Nuclear protein high mobility group box 1 (HMGB1) has emerged as a key inflammatory mediator that is released during sepsis by activated immune cells and necrotic tissue where it functions as a damage-associated molecular pattern (DAMP)^11^. Extracellular HMGB1 interacts with toll-like receptor 4 (TLR4) and the receptor for advanced glycation endproducts (RAGE) to promote chemotaxis and NF-κB signalling. Therapies targeting HMGB1 via molecular inhibitors^12^ or upstream inhibition^13^ has been shown to reduce mortality in sepsis models, and has a wide therapeutic window^14, 15^. Extracellular HMGB1 exists in various structural arrangements dependant on release^11^; for instance necrosis triggers extracellular release of DNA-bound HMGB1^16^, whilst apoptotic cells release HMGB1-containing vesicles^17^. Once in the extracellular space, HMGB1 readily forms complexes with different chemokines, cytokines and bacterial components to instigate differential downstream effects.

Importantly, HMGB1 has been implicated in post-septic immunosuppression through varying redox states of three cysteine residues within the full-length protein. While disulphide HMGB1 exerts pro-inflammatory effects through TLR4 and RAGE^18^, terminally oxidised HMGB1 has roles in the resolution of immune responses, tissue regeneration^18–20^ and the induction of tolerance^21, 22^. In the context of sepsis, HMGB1 may function as a biological ‘switch’ to instigate inflammation resolution and potentially drive immune dysregulation following the acute pro-inflammatory phase. Consequently, a passive immunotherapy approach using anti-HMGB1 polyclonal antibodies may confer additional benefits over that observed with monoclonal therapies by their ability to neutralise multiple epitopes on different HMGB1 forms, thus inhibiting several DAMP-associated functions of HMGB1 at once.

## Results

### Elevated plasma HMGB1 is associated with morbidity and mortality in a cohort of septic shock patients

Previous studies have demonstrated elevated HMGB1 in patients currently with, or recovered from sepsis, with many measurements taken at a minimum of 24 hour intervals^23^. To more closely profile plasma HMGB1 levels in patients during the early phase of septic shock, 17 patients admitted to the intensive care unit (ICU) were enrolled in an observational study where arterial blood was sampled twice daily for the duration of septic shock. Patient characteristics are summarised in Table 1; patients had a median age of 61 and the majority presented with respiratory (35%) or gastrointestinal (29%) sepsis. The age of surviving and non-surviving patients was not significantly different.

**Table 1:** Patient cohort characteristics.

Characteristic	Total (n = 17)	Survivors (n = 9)	Non Survivors (n = 8)
Age^a^	61 (47–68)	55 (45–65)	64.5 (49–77)
Gender	11 female (64%)	7 female (77%)	4 female (50%)
APACHE II Score^a^	18 (15–26.5)	18 (13.5–18.5)	26.5 (16.3–34.8)
Site of infection			
Lung	6 (35.3%)	3 (33.3%)	3 (37.5%)
Gastrointestinal	5 (29.4%)	3 (33.3%)	2 (25%)
Skin	4 (23.5%)	2 (22.2%)	2 (25%)
Other	2 (11.8%)	1 (11.1%)	1 (12.5%)
Positive culture	12 (70.5%)	6 (66.6%)	6 (75%)
G+	3 (17.6%)	1 (11.1%)	2 (25%)
G−	3 (17.6%)	2 (22.2%)	1 (12.5%)
Fungal	1 (5.8%)	0	1 (12.5%)
Mixed	5 (29.4%)	3 (33.3%)	2 (25%)

Unless otherwise stated, data has been represented as: number of patients (percentage of n).

^a^Data represents median (interquartile range).

**Figure 1:**
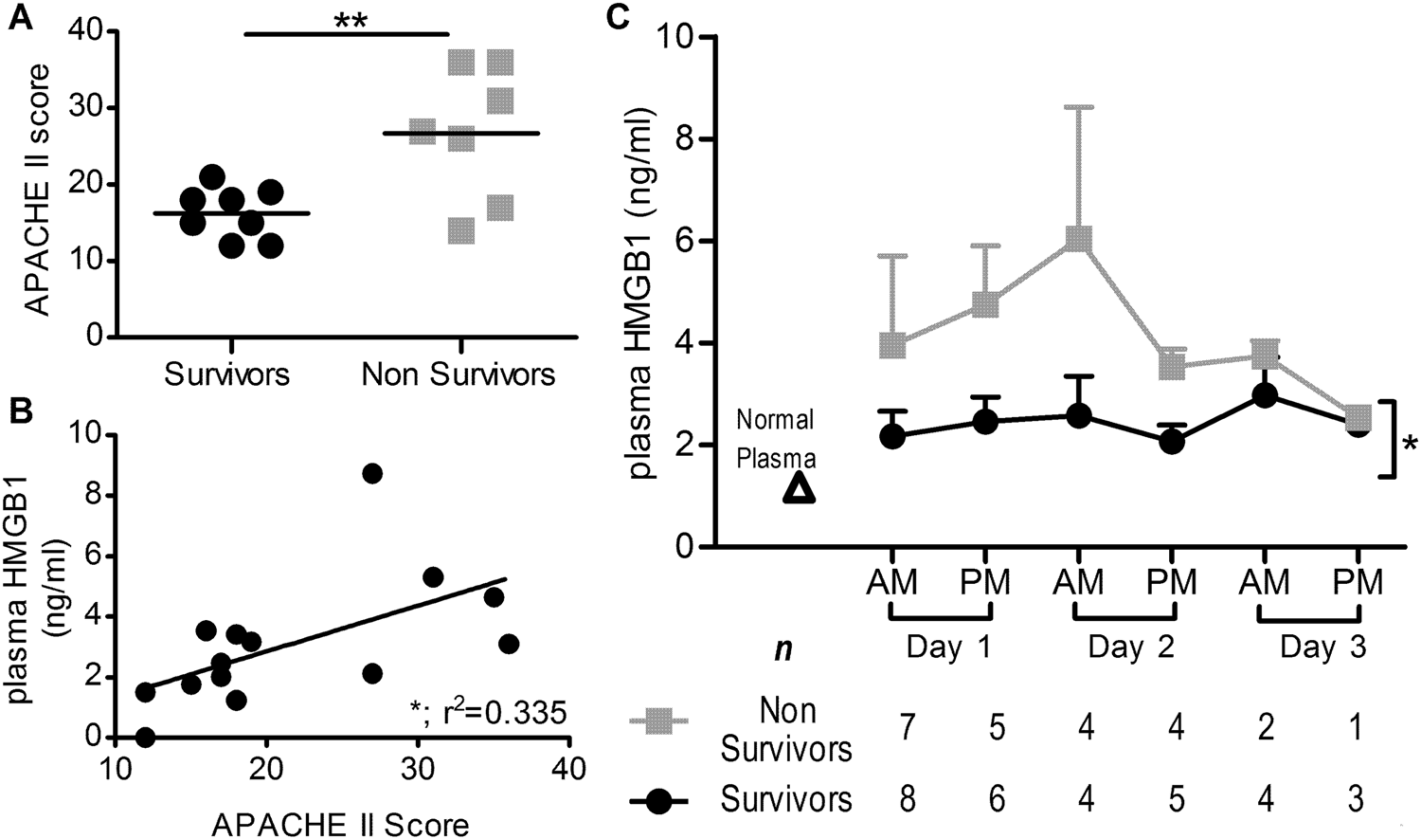
Plasma HMGB1 concentrations and disease severity and mortality in patients with septic shock. Plasma samples from ICU patients with diagnoses consistent with septic shock were taken twice daily (morning and afternoon) for the length of ICU stay. (**A**) APACHE II scores in survivors and non-survivors were compared via two-tailed t test. (**B**) HMGB1 was quantified in patient samples by ELISA and regression analyses performed to compare plasma HMGB1 at 24 hours post ICU admission and APACHE II score. (**C**) Mean kinetic plasma HMGB1 levels in survivors versus non-survivors were compared by paired two-way t-test. Significance is denoted as thus: *P < 0.05, **P < 0.01.

Patients were assigned an acute physiology and chronic health evaluation (APACHE) II sickness severity score within 24 hours of admission to ICU, and kinetic plasma samples taken as described. Non-surviving patients exhibited a significantly higher APACHE II score than surviving patients (Fig. 1A), with HMGB1 levels positively correlated with APACHE II score (Fig. 1B) at 24 hours. Non-survivors also exhibited significantly elevated HMGB1 concentrations overall, with differences most pronounced 24–48 hours after admission, where HMGB1 levels were almost threefold higher in non-surviving patients (2.58 ± 0.76 vs 6.04 ± 2.58 in survivors and non-survivors, respectively; Fig. 1C).

### An exacerbated pro-inflammatory cytokine profile is associated with increased plasma HMGB1 and non-survival during septic shock

**Figure 2:**
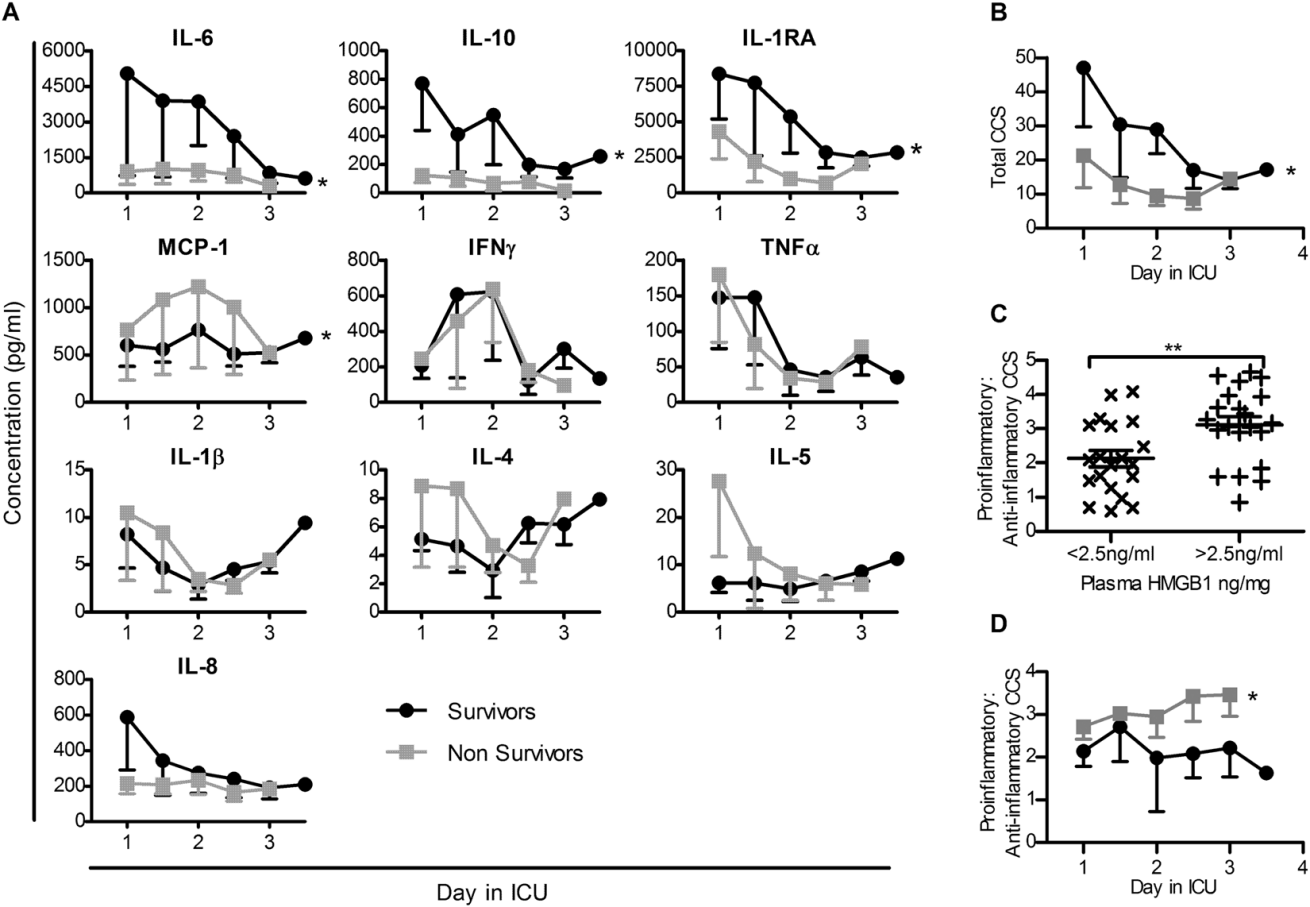
Mortality and elevated HMGB1 are associated with a proinflammatory serum cytokine profile in a cohort of septic shock patients. Plasma samples from septic shock patients were stored and analysed via multiplex analysis. A panel of cytokines (IL-6, MCP-1, IL-10, IFNγ, TNFα, IL-1β, IL-4, IL-5, IL-8 and IL-1RA) was assessed and concentrations calculated from samples run in duplicate. (**A**) Plasma concentrations of individual cytokines between survivors (black) and non-survivors (grey) were compared via paired t-test. Cytokine measurements normalised to the median concentration were used to determine composite cytokine scores (CCS). (**B**) Total CCS indicating the total magnitude of cytokine levels was calculated by summation of all ten normalised cytokine scores and compared between survivors and non-survivors. The ratio of pro-inflammatory (IL-6, MCP-1, IFNγ, TNFα, IL-1β, IL-5 and IL-8) to anti-inflammatory (IL-10, IL-4 and IL-1RA) signalling was compared between samples with a low and high HMGB1 content (**C**) and between survivors and non-survivors (**D**). Plasma cytokine concentrations and ratio between survivors and non-survivors were compared via paired t-test and low vs high HMGB1 content samples were compared via unpaired t-test. Data are depicted as mean ± SEM, with significance is denoted as thus: *P < 0.05, **P < 0.01.

In order to define the cytokine profiles within the septic shock cohort, individual plasma samples were subjected to multiplex cytokine analysis. Assessment of results revealed consistently elevated IL-1RA, IL-6, IL-8 and MCP-1 in septic shock patients compared to published healthy cohorts^24^, with non-survivors exhibiting significantly lower plasma IL-1RA, IL-6 and IL-10, and increased MCP-1 levels compared to survivors (Fig. 2A). Both MCP-1 and IL-1RA were almost two-fold higher in survivors compared to non-survivors in the first 48 hours, with IL-10 also consistently higher in survivors. Notably, IL-6 was five-fold higher in survivors on day 1 compared to non-survivors. Consistent with previous studies, concentrations of TNFα and IL-1β were highest on day 1 in both groups before declining.

The variable efficacy of previously trialled immune modulating therapies such as anti-TNFα has been linked to variations in the inflammatory profile of patients^25^. Thus, clinical targeting of HMGB1 should be informed by analysis of HMGB1 in the context of the septic inflammatory state. In order to evaluate overall inflammatory profiles within the septic shock cohort, composite cytokine scores (CCS) were calculated^3^. Survivors exhibited significantly higher total CCS which decreased over time, while non-survivors exhibited lower total CCS (Fig. 2B). Calculated ratios of pro-inflammatory (IL-6, MCP-1, IFNγ, TNFα, IL-1β, IL-5 and IL-8) to anti-inflammatory (IL-10, IL-4 and IL-1RA) cytokines were significantly higher in plasma samples with elevated HMGB1 (>2.5ng/mL, the median HMGB1 value in analysed samples) compared to those with reduced HMGB1 (Fig. 2C). Furthermore, a significant decrease was observed in inflammatory CCS in survivors compared to non-survivors (Fig. 2D). No individual cytokine significantly correlated with HMGB1 content.

### Ovine anti-HMGB1 pAb therapy improves survival in an in vivo model of murine endotoxaemia

**Figure 3:**
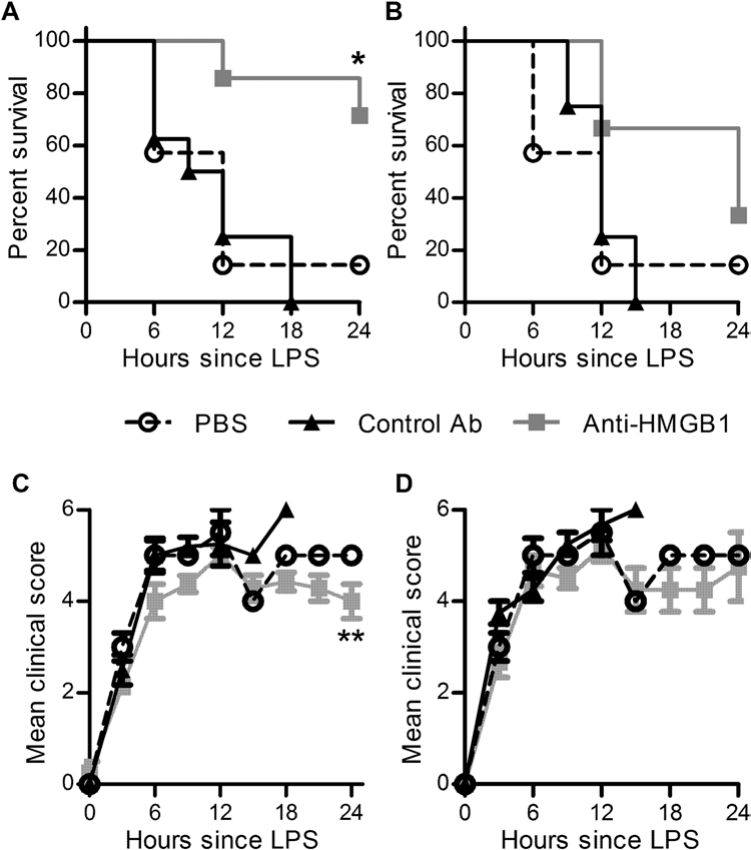
Ovine anti-HMGB1 pAb therapy improves survival in an *in vivo* model of murine endotoxaemia. Adult male and female B6 mice were prophylactically administered 20 mg/kg anti-HMGB1 pAb (*n* = 7), control pAb (*n* = 7) or PBS (*n* = 7) via IP injection one hour before IP injection of 20 mg/kg LPS (A&C). In a therapeutic model, mice were administered 20 mg/kg anti-HMGB1 pAb (*n* = 6), control pAb (*n* = 4) or PBS (*n* = 7) one hour after LPS administration (B&D). Experimental mice were monitored over twenty-four hours for clinical score and euthanised if predetermined humane endpoints were reached. Survival (**A**,**B**) and mean clinical score (**C**,**D**) of groups is represented. Survival curves were compared to controls using the Mantel-Cox test and clinical score data compared to PBS control via two-way ANOVA, with significance denoted as: *P < 0.05, **P < 0.01.

As elevated HMGB1 levels were associated with poor clinical outcomes and dysregulated inflammatory profiles in human patients, a passive immunotherapeutic approach to block HMGB1 was developed and assessed for impact on survival in three murine sepsis models of increasing complexity. Firstly, purified ovine anti-HMGB1 polyclonal antibodies (pAb) were generated^26^ and applied to a simple model of sepsis, lethal endotoxaemia. Mice that received anti-HMGB1 pAbs one hour prior to LPS administration (prophylactic) exhibited a significant survival advantage compared to controls, with a survival rate >70% (Fig. 3A). Groups that received either prophylactic control pAb or PBS displayed expected sickness symptoms and typically required euthanasia within twenty-four hours. In contrast, mice given prophylactic anti-HMGB1 pAbs exhibited significantly fewer symptoms than controls, including surviving mice from the PBS control group (Fig. 3C). Similarly, a trend towards increased survival was observed in anti-HMGB1 pAb-treated mice one hour after LPS administration, with 30% not requiring euthanasia within the experimental period (Fig. 3B). This lower survival rate was evidenced by an increase in the mean clinical score compared to those mice that received prophylactic administration of pAbs (Fig. 3D). Taken together, these results suggest that ovine anti-HMGB1 pAb can increase survival from acute and rapid severe systemic inflammation induced by LPS in this lethal and highly artificial model of sepsis.

### Ovine anti-HMGB1 pAb-treatment improves survival from CLP sepsis

**Figure 4:**
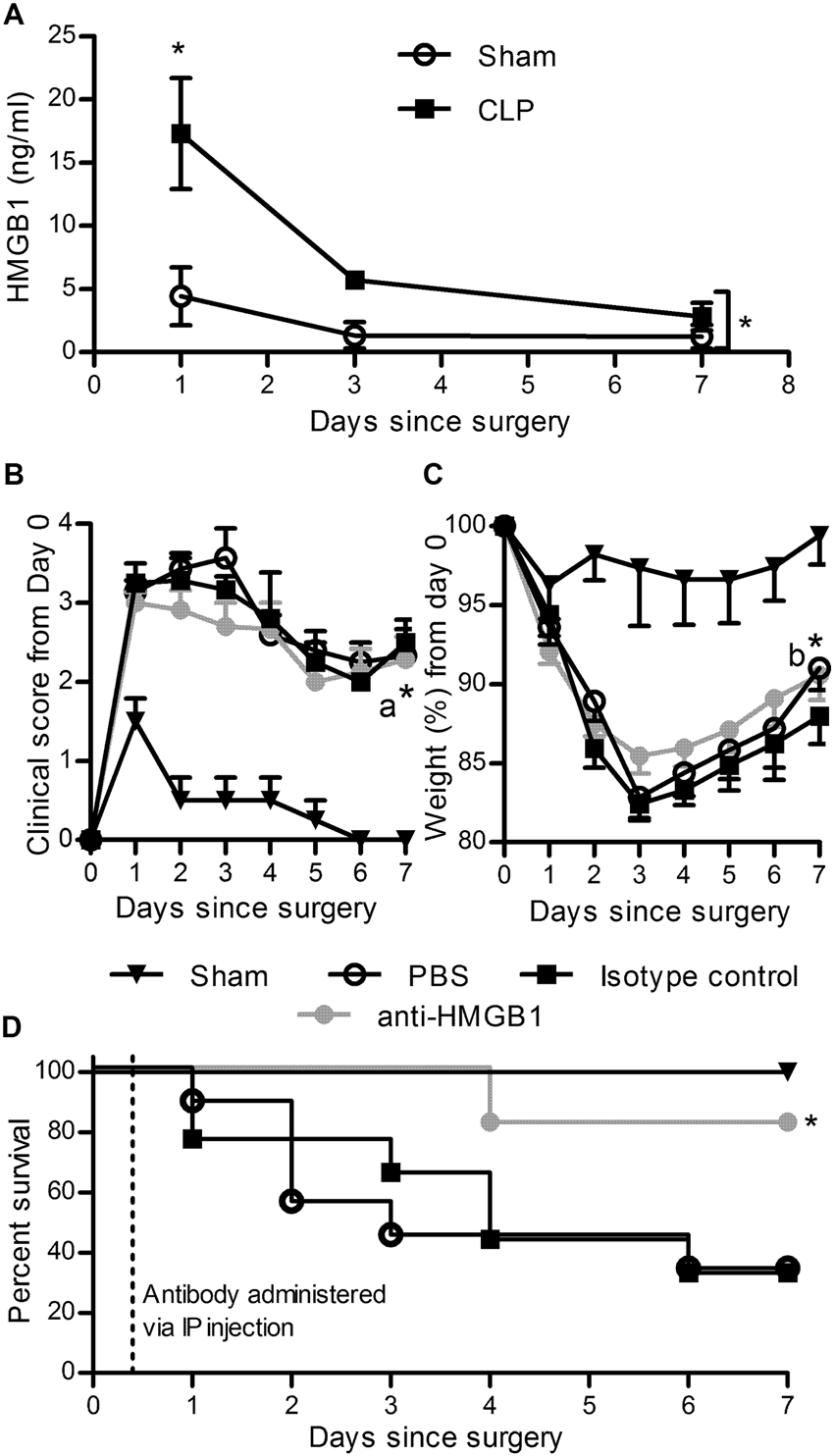
Therapeutic administration of ovine anti-HMGB1 pAb improves survival from CLP sepsis. Mice were subjected to sham-surgery alone (n = 4) or CLP surgery (n = 4) and serum samples from sham or CLP-surgery mice were assayed for HMGB1 via ELISA (**A**). CLP mice (n = 9–11) were administered PBS, control antibody or anti-HMGB1 pAb (25 mg/kg) via IP injection eight hours following surgery. Mice were monitored and euthanised if predetermined humane endpoints were reached. Mean ± SEM of clinical score (**B**) and weight (**C**) and survival curves of the groups (**D**) are depicted. Sequential measurements were compared via two-way ANOVA and survival of anti-HMGB1 treated mice was compared to control groups using the Mantel Cox test with significance denoted as: *P > 0.05, a*P > 0.05 compared against PBS control, b*P > 0.05 compared against control Ab.

While sudden endotoxaemia is a useful model for acute systemic inflammation, sepsis induced by caecal ligation and puncture (CLP) more accurately mimics human sepsis caused by a central focus of infection. It is considered the gold-standard rodent sepsis model as the perforated portion of the caecum forms a necrotic mass within the peritoneal cavity and intermittently releases bacteria^27^. This results in a prolonged period of bacterial challenge, and thus inflammatory cytokine release. A CLP procedure that resulted in 60–70% mortality was developed to assess the therapeutic administration of ovine anti-HMGB1 pAbs. Analysis of serum obtained from mice subjected to CLP or sham surgery (laparotomy only) indicated that the CLP procedure significantly elevated circulating HMGB1 levels at 24hrs, with levels declining thereafter (Fig. 4A). Groups of mice administered PBS or control pAb 8 hours following CLP surgery displayed expected range of symptoms of ill health (hunched posture, ruffled fur, a reluctance to move and weight loss) which were not exhibited by mice undergoing sham surgery (Fig. 4B). Mice treated with anti-HMGB1 pAbs 8 hours after CLP exhibited consistently lower mean clinical scores compared to PBS-treated mice. Overall weight loss was significantly reduced in anti-HMGB1 pAb-treated groups compared to control Ab-treated groups (Fig. 4C), and together with improved clinical score, resulted in significantly increased survival (Fig. 4D). Collectively, these results indicated that therapeutic anti-HMGB1 pAb treatment can treat septic mice and promote recovery from clinical symptoms to increase survival.

### Ovine anti-HMGB1 pAb-treated mice display cytokine profiles associated with improved clinical outcomes in the early phase of CLP sepsis

**Figure 5:**
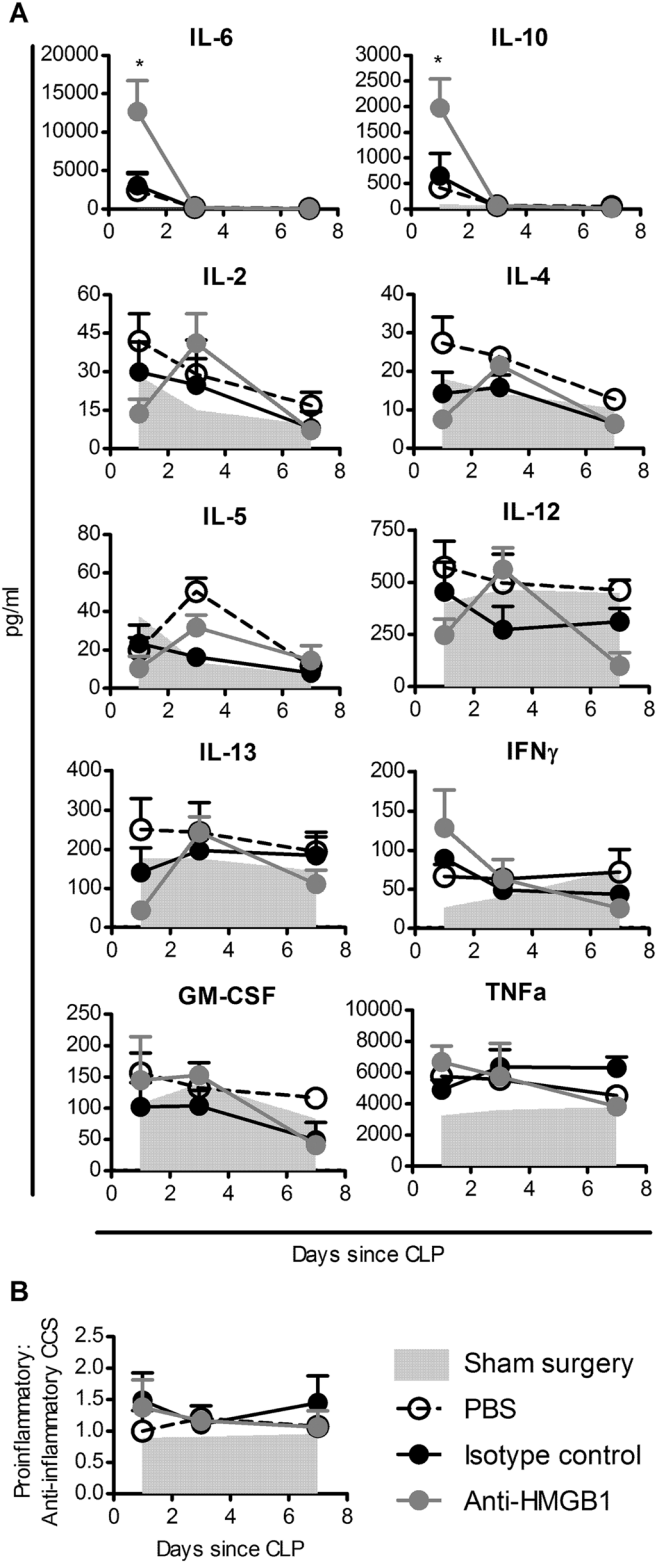
Ovine anti-HMGB1 pAb-treated mice display cytokine profiles associated with improved clinical outcomes in the early phase of CLP-induced sepsis. Blood samples from mice (n = 3–5) subjected to sham-surgery (pale grey area) or CLP-induced sepsis and treated with PBS (unfilled symbols), control pAb (25 mg/kg; black symbols) or anti-HMGB1 pAb (25 mg/kg; grey symbols) were analysed for cytokine content using a multiplex platform. Mean + SEM of individual cytokine measurements are depicted. (**A**). Proinflammatory (IL-2, IL-5, IL-6, IL-12, IFNγ, GM-CSF and TNFα) to anti-inflammatory (IL-4, IL-10 & IL-13) CCS were calculated for each sample, mean + SEM group CCS normalised to median values are depicted in (**B**). Levels of cytokines were compared using two-tailed T-tests, with significance denoted as thus: *P < 0.05.

In order to determine the effect of HMGB1 neutralisation on the inflammatory profile in CLP-induced sepsis, serum samples were obtained on day 1, 3, and 7 post surgery and assessed for levels of pro- and anti-inflammatory cytokines. Induction of sepsis by CLP resulted in early 1.5–20-fold increases in IL-6, IL-10, IFNγ and TNFα over that induced by sham surgery. Treatment with anti-HMGB1 pAbs 8 hours post-surgery resulted in a further significant five-fold increase in IL-6 and a four-fold increase in IL-10 in anti-HMGB1 mice at day 1 after CLP compared to groups that received PBS or isotype control pAbs (Fig. 5A). Additionally, cytokine expression in anti-HMGB1 pAb treated animals displayed a delayed response, with reduced levels of IL-2 IL-4, IL-12 and IL-13 observed 1 day after CLP compared to controls. These cytokine levels then increased to control group day 1 levels by day 3, and diminished thereafter. Pro-inflammatory:anti-inflammatory CCS calculated for the panel of cytokines tested were not significantly altered between groups (Fig. 5B). These data indicate that anti-HMGB1 pAb treatment altered the early-phase inflammatory response generated by CLP-induced sepsis, which ultimately improved survival.

### Ovine anti-HMGB1 pAb-treatment during CLP-induced sepsis reduces susceptibility to secondary *P. aeruginosa* infection

**Figure 6:**
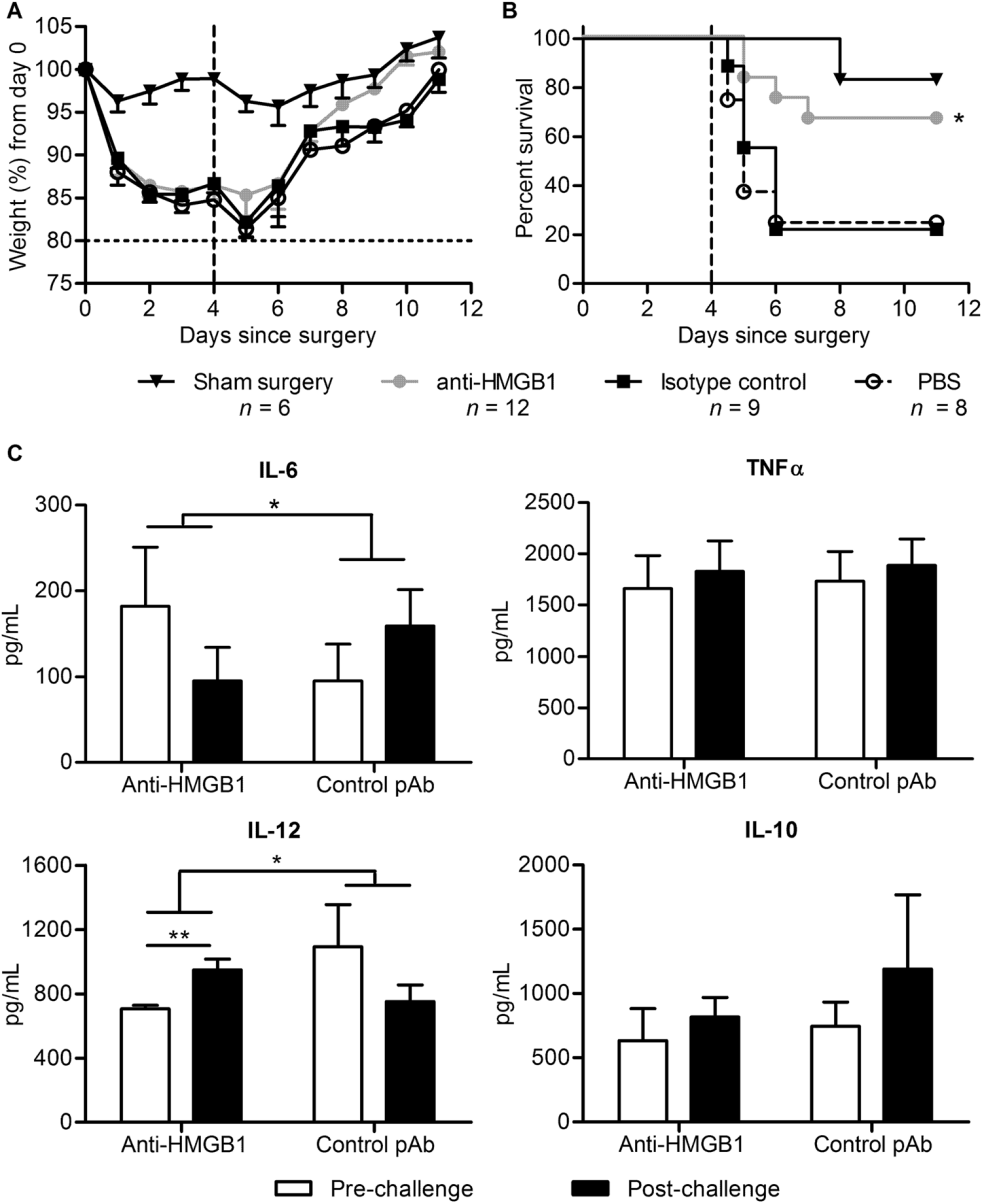
Prior anti-HMGB1 pAb treatment alters the inflammatory response and survival to secondary bacterial challenge in mice that have survived CLP-induced sepsis. Mice (n = 6–12) were subjected to sham-surgery or mild CLP and allowed to recover before IN instillation of *P*. *aeruginosa* (2.5 × 10^7^ CFU) or PBS on Day 4 (indicated by dashed line). Mice were treated 8 hours following surgery with anti-HMGB1 or control pAbs (20 mg/kg) or PBS and administered maintenance pAb doses (10 mg/kg) or PBS on days 3 and 6. Mice were monitored for disease symptoms and euthanised at predetermined humane endpoints. Mean - SEM weight loss (**A**; humane weight loss endpoint indicated by horizontal dashed line) and survival (**B**) and of mice are depicted. Serum samples collected from mice (*n* = 4–5) immediately prior to bacterial challenge and 24 hours after challenge were analysed for IL-6, IL-10, IL-12 or TNFα content via ELISA (**C**). Survival curves were compared via Mantel-Cox analysis, and changes in cytokines before and after treatment were compared via paired t-test. Cytokine changes between groups were compared via two-way matched pairs ANOVA, with significance denoted as thus: *P < 0.05, **P < 0.01.

While anti-HMGB1 pAb treatment increased survival in the short term after CLP, it was necessary to determine if surviving mice exhibited post-septic immunosuppression. In order to assess susceptibility of mice to a secondary infection, a modified CLP procedure which induced weight loss and 30% lethality by day 3 was performed. Survivors subsequently received an intranasal challenge with live *P*. *aeruginosa*. Mice subjected to initial sham-surgery exhibited transient weight loss after surgery and mild symptoms of infection after bacterial challenge (Fig. 6A), with only 1/6 not surviving (Fig. 6B). In contrast, septic PBS or isotype control pAb-treated mice exhibited sharp clinical decline, including weight loss, after bacterial challenge (Fig. 6A). The majority reached predetermined humane endpoints within 12–48 hours (Fig. 6B) clearly indicating an inability to control a secondary infection. Treatment of CLP-induced sepsis with anti-HMGB1 pAb however, left these mice with significantly higher survival rates after secondary bacterial challenge than either control groups (Fig. 6B). Whilst weight loss in this group was not significantly different from controls, anti-HMGB1 pAb-treated mice returned to normal weight earlier than survivors from untreated groups, suggestive of a faster time to recovery following bacterial challenge (Fig. 6A).

To assess the effect of anti-HMGB1 pAb therapy on IL-6, IL-10, TNFα and IL-12 cytokine profiles in this secondary challenge model, blood samples were taken immediately prior to administration of *P*. *aeruginosa* on day 4, and then again, 24 hours later. No significant difference in IL-6, IL-10 or TNFα concentrations between pre- and post-challenge samples in either pAb-treated group was observed; however anti-HMGB1 pAb-treated mice exhibited a significant increase in IL-12 serum concentration post-challenge which was not observed in control pAb-treated mice (Fig. 6C). Analysis of cytokine expression pre- and post-challenge between groups via two-way matched-pairs ANOVA revealed that anti-HMGB1 pAb-treatment significantly altered IL-6 and IL-12 responses to bacterial challenge. Taken together, these results suggest anti-HMGB1 pAb treatment improves survival from secondary infection following CLP-induced sepsis, potentially via alterations in cytokine responses.

### Anti-HMGB1 pAb-treatment during CLP-induced sepsis alters macrophage and dendritic cell cytokine expression profiles in surviving mice

**Figure 7:**
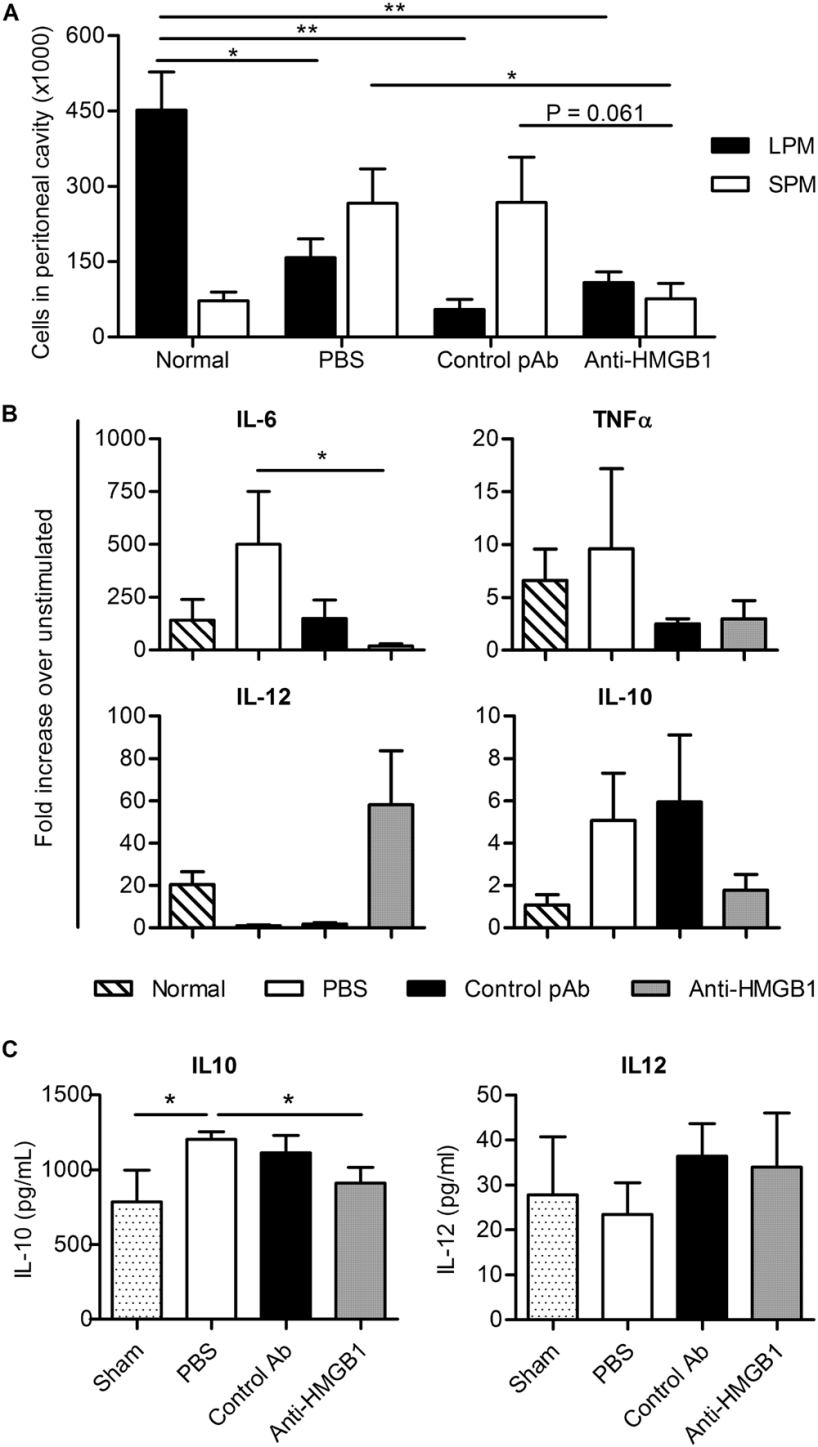
Anti-HMGB1 pAb treatment alters macrophage and DC cytokine expression post survival from CLP-induced sepsis. Peritoneal cells were harvested four days following CLP surgery and treatment with either PBS (n = 4), control ovine pAb (n = 4) or anti-HMGB1 pAb (n = 5) and evaluated via flow cytometry. Frequency of large peritoneal macrophages (LPM; CD11b^hi^, F4/80^hi^, Gr-1^+^) and small peritoneal macrophages (SPM; CD11b^lo^, F4/80^lo^, Gr-1^−^) compared to normal mice (n = 2) are expressed (**A**; Mean + SEM). Adherent macrophages from peritoneal lavages were stimulated with LPS (100 ng/mL) for 18 hours and IL-6, IL-10, IL-12 and TNFα content in the supernatant was evaluated via ELISA. Mean + SEM expression for each treatment relative to unstimulated wells is expressed (**B**). Bone marrow cells harvested from Day 4 septic or sham-surgery mice were cultured in the presence of GM-CSF to generate bone-marrow derived dendritic cells (BMDCs). Harvested BMDC’s were plated and cultured in the presence of LPS (100 ng/mL); Mean + SEM IL-10 and IL-12 cytokine content of resulting supernatants is expressed (**C**). Mean values of individual groups were compared via two-tailed t tests, with significance denoted as thus: **P < 0.01, *P < 0.05.

The increased susceptibility of sepsis survivors to secondary infection has been linked to post-septic immunosuppression characterised by functional exhaustion of lymphocytes, immune cell apoptosis and epigenetic changes within immune cells, including macrophages. Two distinct subsets of macrophages with differential capacity to respond to stimuli exist within the peritoneum. Large peritoneal macrophages (LPM) are most abundant at resting state and perform roles in homeostasis and maintenance^28^, while small peritoneal macrophages (SPM) rapidly accumulate in the peritoneum following an inflammatory stimulus^29^, and are implicated in conferring inflammatory function in disease^29, 30^. To investigate the effect of anti-HMGB1 pAb treatment on the peritoneal macrophage population during sepsis, peritoneal cells were harvested from treated and untreated mice 4 days after CLP-induced sepsis. Flow cytometric analysis was performed to determine the frequency of LPM (CD11b^hi^, F4/80^hi^, Gr-1^+^) and SPM (CD11b^lo^, F4/80^lo^, Gr-1^−^) in the peritoneal compartment (Fig. 7A). The frequency of LPM was highest in normal mice not subjected to surgery, with all CLP groups regardless of treatment exhibiting significantly lower total numbers of LPM. In contrast, an increase in SPM numbers was observed only in CLP groups treated with PBS or isotype control pAb, with anti-HMGB1 pAb treated mice harbouring numbers of SPM equivalent to that observed in normal mice, suggesting that neutralisation of HMGB1 inhibited the influx of inflammatory macrophages to the peritoneal cavity.

To determine whether the macrophage populations present 4 days after sepsis induction exhibited altered functional responses, peritoneal macrophages were harvested 4 days after induction of sepsis, stimulated with LPS *ex vivo* for 18 hours and supernatant analysed for IL-6, TNFα, IL-10 and IL-12. It was clear that anti-HMGB1 treatment of septic mice resulted in macrophages that expressed levels and patterns of cytokines that were more similar to that expressed by peritoneal macrophages obtained from normal mice than macrophages from either PBS or isotype control pAb treated septic mice (Fig. 7B).

Susceptibility to *P*. *aeruginosa* following CLP-induced sepsis has been linked to reduced IL-12 and increased IL-10 production by dendritic cells (DCs). Therefore, to determine if anti-HMGB1 pAb treatment impacted DC function, bone marrow derived dendritic cells (BMDCs) were generated from sham-surgery, PBS treated, control pAb-treated or anti-HMGB1 pAb-treated mice four days after CLP-surgery. After 7 days culture in the presence of GM-CSF, semi-adherent BMDCs were stimulated with LPS and assayed for IL-10 and IL-12 production. Though IL-12 production was not significantly different between treatment groups, results clearly indicated that BMDC generated from anti-HMGB1 treated mice secreted a similar amount of IL-10 to that observed from sham surgery, non-septic mice, which suggested that neutralising HMGB1 in the context of an initial inflammatory response can alter subsequent responses.

## Discussion

Clinical and preclinical studies have implicated HMGB1 as a mediator of sepsis pathogenesis and mortality, and thus HMGB1 represents a novel candidate for passive immune targeting in sepsis. Our studies presented here indicate that administration of anti-HMGB1 pAb to a murine model of severe sepsis alters the pattern of cytokine signalling to a more favourable profile, improves survival, and protects against the development of post-septic immunosuppression. The association between plasma HMGB1 and morbidity and mortality within the septic shock patient cohort is consistent with findings from several previous studies where HMGB1 was found to be elevated in patients with more severe illness^31–34^. A 2008 study of severe sepsis patients revealed a significant correlation of plasma HMGB1 with SOFA score in the most severely ill subgroup of patients, however HMGB1 was not found to be different between all survivors and non-survivors within the cohort^35^. An earlier study had demonstrated a significant decrease in plasma HMGB1 in non-survivors using a western-blot method however this result was not recapitulated using an alternate western-blot protocol^23^. In the current study, HMGB1-specific ELISA was employed to measure patient and murine samples. It has been postulated that while ELISA may be a more sensitive method for detection of plasma biomarkers, HMGB1 binding proteins or other masking factors may complicate HMGB1 detection in these assays^23, 34^. Consequently, further studies are required to determine the most useful method to effectively measure bioavailable HMGB1.

Individual plasma cytokine levels are not considered good prognostic indicators in clinical practice due to the inherent variability seen in sepsis^36, 37^, however as expected the concentrations of several cytokines were different between survivors and non-survivors. For example, IL-6 was higher in survivors, a finding at odds with previous studies^38^, although in a manner similar to this study, elevated IL-6 levels in the first 48 hours from sepsis onset in survivors has also been reported^39^. Importantly, only septic shock patients were sampled within the current study, which comprise a unique sub-population within sepsis patients. Non-survivors exhibited rapid increases in IL-4 and IL-1RA from day 2, and concurrent pro-inflammatory increases in TNFα and IL-1β which were not statistically significant. The profile of overall cytokine release may be a more valuable measurement than individual cytokine concentrations. Hence in this study, cytokine measurements were used to calculate pro-inflammatory:anti-inflammatory ratios for each sample. Non-survivors had significantly increased pro-inflammatory capacity which also increased over time compared to survivors. Increased plasma HMGB1 was also associated with an enhanced pro-inflammatory profile supporting the notion of HMGB1 as a pro-inflammatory cytokine. Non-survivors exhibited reduced overall cytokine signalling over the sampling period. Together, these observations may indicate that non-survivors are not able to balance early pro-inflammatory signalling leading to increased pathology and lethality. Additionally, measured concentrations of anti-inflammatory cytokines IL-4, IL-10 and IL-1RA increased at later time points in the surviving cohort, which may indicate effective regulation of inflammation. The timeframe of sampling in this study was too short to deduce long-term signalling patterns, however it can be speculated that assessment of HMGB1 concentration and pro-inflammatory:anti-inflammatory cytokine ratio may aid in monitoring clinical course. The broader availability of efficient multiplexed plasma analysis technology simplifies the monitoring of these parameters in a clinical setting.

The observations from the patient study support development of a septic shock treatment targeting HMGB1. It was hypothesised that HMGB1 blockade may be more efficacious in the clinic than targeting pro-inflammatory cytokines, as HMGB1 exhibits both pro-inflammatory and anti-inflammatory activities and has a wider therapeutic window^11, 22^. In a short-term model of murine endotoxaemia, anti-HMGB1 pAb administration increased survival when administered prophylactically, providing proof-of-principal that anti-HMGB1 pAb could reduce inflammation *in vivo*. As an example of a more complex septic disease, the impact of anti-HMGB1 pAb on septic inflammatory profile was assessed in the CLP model. Mice subjected to CLP-surgery had significantly higher serum HMGB1 than sham-surgery controls, though HMGB1 release was earlier and more transient than in patient groups, which may reflect the kinetics of sepsis induced by CLP. Anti-HMGB1 pAb treatment increased survival in this model compared to control groups. Treated mice also had fewer clinical signs of disease and reduced weight loss than control groups, indicating that HMGB1 blockade reduced morbidity and mortality from CLP sepsis, a result in agreement with previous studies^40, 41^. Analysis of cytokine profiles following treatment revealed significant elevations in IL-6 and IL-10 in anti-HMGB1 pAb-treated mice 24 hours following surgery, which were both associated with survival in the septic shock patient cohort. Previous studies have implicated IL-10 as an important regulator of early inflammation which delays the onset of septic shock^9^. In the context of the current findings, the observed increase in IL-10 in survivors and anti-HMGB1 pAb-treated mice may serve to modulate early pro-inflammatory pathways and reduce lethality.

Patients that survive the early inflammatory phase of sepsis may become immunosuppressed and contract secondary infections. While anti-HMGB1 pAb therapy increased survival in simple endotoxaemia and CLP sepsis, it was unknown whether anti-HMGB1 could potentiate immunosuppression and increased susceptibility to secondary infection. To investigate this, a ‘double-hit’ model of CLP-induced sepsis followed by bacterial challenge was employed, where sepsis survivors exhibit increased susceptibility to *Pseudomonas* challenge^42^. Anti-HMGB1 pAb-treated septic mice had a survival advantage compared to control mice following secondary infection and treatment was associated with improved antimicrobial responses as mice exhibited increased IL-12 and decreased IL-6 following infection. As post-septic immunosuppression is associated with altered innate immune cell function^43, 44^, macrophages in the peritoneal cavity from CLP mice were evaluated at day 4 following CLP. Anti-HMGB1 pAb-treated mice exhibited decreased numbers of SPMs within the peritoneal cavity, which may be a result of a less severe disease course as SPMs migrate to the peritoneal cavity in response to inflammatory challenge^28^. Importantly, peritoneal macrophages from anti-HMGB1 pAb-treated mice displayed significantly decreased IL-6 and increased IL-12 expression after *in vitro* LPS stimulation compared to controls. Recently, DC dysfunction has been implicated in the double-hit model where reduced IL-12 and increased IL-10 production promote susceptibility to secondary infection^45^. Here, BMDC generated from anti-HMGB1 pAb-treated CLP mice expressed lower quantities of anti-inflammatory IL-10 when compared to BMDC from untreated mice indicating that the immunosuppressive phenotype of dendritic cells was altered by HMGB1 inhibition, however no such differences were observed for IL-12.

While the role of HMGB1 in the development of immunosuppression and immunoparalysis in sepsis is poorly understood, several potential mechanisms have been described that may contribute to the results described here. The observed phenotype of peritoneal macrophages from septic mice likely represents a partial tolerance to LPS, as it has been shown that HMGB1 directly induces LPS tolerance in macrophages^46^, in addition to other pro-inflammatory cytokines. Thus, the observed effect of anti-HMGB1 pAb treatment on macrophage function may be mediated by both direct blockade of HMGB1 signalling on macrophages, and by an altered inflammatory milieu. As the administration of ‘naïve’ DCs can reverse post-septic disease susceptibility in the double-hit model^47^, DC function is clearly central to the development of septic immunosuppression. In the current study, anti-HMGB1 pAb treatment altered the response of BMDCs to LPS stimulation with less IL-10 secreted after LPS stimulation than from BMDCs derived from untreated septic mice. While HMGB1 is necessary for DC maturation^48^, excessive HMGB1 release may contribute to abnormal maturation of DCs to promote Th2 responses^49^ and consequently anti-HMGB1 pAb may alter DC function by interfering with these pathways.

It has been shown here that anti-HMGB1 pAb are efficacious in experimental sepsis, they alter inflammatory profiles associated with clinical lethality and do not promote immunosuppression. Though many immune-modulating therapies have been trialled in sepsis, none have exhibited significant clinical benefit over current standard-of-care^50^. In this study, inflammatory profiles in patients were heterogeneous, thus a more effective approach to treating sepsis may involve delivering therapies reflective of their immune status. Therapies that target master immune regulators could serve to first reduce the severity of cytokine deregulation in early sepsis before targeting specific pro- or anti- inflammatory cytokines to reverse pathogenic immune signalling. As such, HMGB1-blockade may aid in dampening the magnitude of inflammatory signalling in sepsis and leave patients more responsive to further therapeutic immunomodulation. These results demonstrate that HMGB1 targeted therapeutics offer potential for further development for the treatment of clinical sepsis and septic shock.

## Methods

### Ethics statement

Human studies were approved by the Royal Adelaide Hospital Research Ethics Committee and accepted by The University of Adelaide and University of South Australia Human Research Ethics Committees under the National Mutual Acceptance system. All procedures relating to the enrolment, sampling and analysis of patients conformed to the provisions of the National Statement on Ethical Conduct in Human Research (2007), Australian National Health and Medical Research Council, with informed consent obtained from all participants or next of kin.

All animal studies were conducted with dual approval from The University of Adelaide Animal Ethics Committee, and the University of South Australia Animal Ethics Committee, and followed institutional and national ethical guidelines according to the principles outlined in the ‘Australian code for the care and use of animals for scientific purposes (2013)’.

### Patient recruitment and clinical study

To profile the presence of serum HMGB1 in patients during the early phase of septic shock, a prospective cohort study was performed on septic shock patients admitted to the Royal Adelaide Hospital (RAH) ICU for a one year period (December 2012 - December 2013). Consecutive patients ≥18 years old admitted to the ICU with septic shock were identified by clinical staff, with septic shock defined as a diagnosis consistent with sepsis (including pneumonia, urinary tract infection and peritonitis) in addition to hypotension (MAP < 70 mmHg) despite adequate fluid resuscitation (>500 mL fluid administered), adequate intravascular volume status (Pulmonary arterial wedge pressure >12 mmHg or Central Venous Pressure >8 mmHg) and the need for vasopressors. Vasopressors included any infusion of noradrenalin and/or adrenalin. Informed consent was sought from patients or next-of-kin as appropriate, and all samples obtained from patients were de-identified prior to storage and analysis. Patients on steroid therapy were excluded from the study, with the remaining patient cohort characteristics summarised in Table 1. Arterial blood was collected from patients into sodium citrate tubes on the first morning after admission (1–12 hours post admission) and every morning and afternoon thereafter for as long as haemodynamic intervention was required or a maximum of 7 days. Blood samples were centrifuged (10 minutes, 1,000 × g) and the plasma aliquoted and frozen (−80 °C). Plasma samples were analysed for HMGB1 content by ELISA (Shino-Test, IBL) according to manufacturer’s instructions, with samples diluted 1 in 2 in assay buffer and a standard curve ranging from 80 ng/ml to 300 pg/ml.

### Ovine polyclonal anti-HMGB1 production

Ovine anti-HMGB1 polyclonal antibodies (pAbs) were generated via immunisation of sheep with purified recombinant HMGB1 in Freund’s adjuvant and purified as previously described^26^. Total anti-HMGB1 and control IgG was purified via Protein G chromatography. Purified pAbs were buffer exchanged to PBS and sterile filtered through a 0.22 µm membrane before freezing in concentrated stocks (5–10 mg/ml). Ovine anti-HMGB1 pAbs were evaluated *in vitro* and *in vivo* as previously described^26^.

### Endotoxin model of toxic shock

Endotoxaemia was induced in 6–8 week old C57Bl/6 mice by IP injection of 20 mg/kg 0111:B4 LPS (Sigma) which caused all animals to reach humane endpoints requiring euthanasia in 12–18 hours. Animals were monitored for signs of toxicity including hunched posture (score 0–2), ruffled coat (score 0–2), diarrhoea (score 0–2), dehydrated eyes (score 0–2), reduced temperature (score 0–1) and reluctance to move (score 0–2) and a cumulative clinical score of 6 or above indicated a requirement for euthanasia. Ovine pAbs (25 mg/kg) or PBS were administered via IP injection either one hour before (prophylactic) or one hour after (therapeutic) LPS administration. The dose of pAbs was determined from empirical trials reported in previous studies^26^.

### Caecal ligation and puncture (CLP) model of sepsis

Aged male 10–12 week old C57Bl/6 mice were weighed, anaesthetised via isoflurane inhalation and their abdomen shaved and disinfected with Betadine solution. Caecal ligation and puncture (CLP) surgery was performed as previously described^51^. Briefly, 7 mm of the tip of the caecum was ligated using 4.0 silk sutures and the tip pierced twice with a 21G needle to produce two punctures. The laparotomy was closed using 4.0 silk sutures and the outer skin closed with 6.0 prolene sutures. Mice were administered subcutaneous Buprenorphine (0.05 mg/kg), Enrofloxacin (5 mg/kg) and saline (1 mL) and allowed to recover from anaesthetic in a pre-warmed cage before transfer to individual cages. Mice were monitored 1–3 times daily for signs of clinical disease and euthanised upon reaching pre-determined humane endpoints, which included a clinical score of 5 or 20% weight loss from surgery day. Mice were injected with saline (1 mL; SC) and provided with moistened food daily. This protocol resulted in >60% of mice reaching humane endpoints requiring euthanasia and is considered a septic syndrome of moderate severity. Treatment with anti-HMGB1 or control pAb (25 mg/kg), or saline was performed 8 hours following surgery to randomly selected mice via intraperitoneal injection. Blood samples for cytokine analysis were obtained on day 1, 3 and 7 post surgery from experimental groups via facial vein bleeds.

### Microbiologic preparation and double-hit sepsis model

To determine if treated mice exhibited reduced post-septic immunosuppression, treated and untreated mice were subjected to a less severe one-puncture CLP surgery and challenged with live *Pseudomonas aeruginosa* four days later. A single bacterial colony grown from a frozen stock of *P*. *aeruginosa* (ATCC 27853) was suspended in fresh TSB and grown overnight at 35 °C with constant shaking. Bacteria pelleted from the broth culture were washed and resuspended in PBS and adjusted to an optical density of 0.5 A_600nm_ as measured on a spectrophotometer. A colony count of ~1 × 10^9^ CFU/mL was confirmed with serial dilution onto TSB agar. Aged male 10–12 week old C57Bl/6 mice were subjected to sham surgery or a modified CLP procedure, where a 5 mm section of caecum was ligated and only one puncture was performed with a 23 G needle. Supportive medications were provided and monitoring was performed as described above and anti-HMGB1 pAbs or control treatments (25 mg/kg) were administered 8 hours following surgery and every 3 days for the duration of the study. On day 4, 96 hours after modified CLP surgery, *P*. *aeruginosa* inoculum (25 µL) was administered via intranasal inhalation under anaesthesia. Mice were monitored as above and euthanised according to pre-determined humane endpoints. For cytokine analysis before and after bacterial challenge, a separate cohort was subjected to modified CLP and bled pre-challenge on day 4 via facial vein bleed, and post-challenge via terminal bleed; these mice were not included in survival analysis.

### Isolation and stimulation of peritoneal macrophages

Peritoneal lavage was performed on humanely killed mice with 3 × 2.5 mL ice-cold endotoxin free PBS supplemented with 1% fetal calf serum. Peritoneal cells were pelleted by centrifugation (300 xg, 5 min) and resuspended in 250 µL red blood cell lysis buffer before incubation (5 min, room temperature). Cells were washed twice with warm RPMI medium supplemented with 10% fetal calf serum, 100 U/mL penicillin and 100 μg/mL gentamycin (cRPMI), and plated at 2 × 10^5^ cells/well (400 µL) in duplicate in 48-well plates. Plates were incubated for 90 min at 37 °C 5% CO_2_ to allow macrophages to adhere. Supernatants and non-adherent cells were subsequently removed, and adherent cells washed with endotoxin-free PBS before incubation with or without LPS (1 μg/mL) in 250 µL cRPMI for 18 hours. Supernatants were subsequently harvested and stored at −20 °C for analysis.

For macrophage analysis of peritoneal lavage cells, cells were washed and stained with Fc Block (anti-CD16/CD32) (1:100), anti-F4/80 PE (1:100), anti-CD11b AF488 (1:100) and anti-Gr-1-biotin (1:100) with streptavidin-APC. Cells were analysed on a three-laser BD FacsCANTO II Flow Cytometer. Doublets were excluded by gating on FSC-A/FSC-H, and viable cells were used for analysis.

### Generation and stimulation of bone marrow-derived dendritic cells (BMDC)

Bone marrows were harvested and used to generate BMDC using GM-CSF sourced from B16-GMCSF conditioned medium. Briefly, bone marrow was flushed from the tibia and femur bones of B6 mice and homogenised via passage through 19G, 21G and 23G needle bores and red blood cells were lysed. Resulting bone marrow cells were counted plated at a density of 5 × 10^5^ cells/mL and cultured for 7 days in complete RPMI medium supplemented with 10% FBS and 10% B16-GMCSF conditioned medium. Media volume was replenished by 50% on Day 3 and Day 5. On day 7, lightly adherent DCs were harvested via gentle pipetting and plated in duplicate wells of 48-well-plates at 2 × 10^5^ cells/mL in the presence of LPS (100 ng/mL). Cell-free supernatants were harvested 18 hours later and frozen for cytokine analysis.

### Cytokine analysis

Cytokines present in human plasma and mouse serum were analysed via BioPlex multiplex platform (BioRad MagPix) according to the manufacturer’s instructions. Cytokines to be measured from human plasma were selected from clinical studies of multiplex cytokine analysis in sepsis patients^52, 53^ and included IL-1β, IL-1RA, IL-4, IL-5, IL-6, IL-8, IL-10, interferon gamma (IFNγ), monocyte chemotactic protein 1 (MCP-1) and TNFα. Measured murine cytokines were based on a Th1/Th2 murine panel and included IL-2, IL-4, IL-5, IL-6, IL-10, IL-12 (p70), IL-13, IFNγ, GM-CSF and TNFα. All samples were run in duplicate using a one-in-five sample dilution and concentrations calculated from a standard curve using Bio-Rad Manager MP Software. Composite cytokine scores (CCS) expressing the ratio of pro-inflammatory to anti-inflammatory cytokines was calculated based on a previously published method^3^. Briefly, human cytokine concentrations were first normalised to the median measurement and CCS was calculated by dividing the sum of normalised pro-inflammatory cytokine values (IL-6, MCP-1, IFNγ, TNFα, IL-1β, IL-5 and IL-8) by the sum of normalised anti-inflammatory cytokine values (IL-10, IL-4 and IL-1RA). Murine CCS were similarly determined using proinflammatory cytokine (IL-2, IL-5, IL-6, IL-12, IFNγ, GM-CSF and TNFα) and anti-inflammatory cytokine (IL-4, IL-10 & IL-13) expression.

Concentrations of IL-6, IL-10, IL-12 and TNFα in culture supernatants were measured via ELISA using commercially available antibodies (eBioscience).

### Statistical analysis

Analysis was performed using GraphPad Prism V3.0 software. Survival curves were analysed via Mantel-Cox testing. Sequential measurements over time were compared between groups using Two-Way ANOVA and individual time points were compared using two-tailed t-tests, with P < 0.05 considered statistically significant.

### Availability of data and materials

The datasets collected during the current study are available from the corresponding authors on reasonable request.
